# The Challenges of Using Horses for Practical Teaching Purposes in Veterinary Programmes

**DOI:** 10.3390/ani6110069

**Published:** 2016-11-11

**Authors:** Gabriella Gronqvist, Chris Rogers, Erica Gee, Charlotte Bolwell, Stuart Gordon

**Affiliations:** Massey Equine, Institute of Veterinary, Animal and Biomedical Sciences, Massey University, Private Bag 11-222, Palmerston North 4442, New Zealand; C.W.Rogers@massey.ac.nz (C.R.); E.K.Gee@massey.ac.nz (E.G.); C.Bolwell@massey.ac.nz (C.B.); S.J.G.Gordon@massey.ac.nz (S.G.)

**Keywords:** horse, equine handling, equine welfare, human-equine interaction

## Abstract

**Simple Summary:**

Veterinary students often lack previous experience in handling horses and other large animals. This article discusses the challenges of using horses for veterinary teaching purposes and the potential consequences to student and equine welfare. The article proposes a conceptual model to optimise equine welfare, and subsequently student safety, during practical equine handling classes.

**Abstract:**

Students enrolled in veterinary degrees often come from an urban background with little previous experience in handling horses and other large animals. Many veterinary degree programmes place importance on the teaching of appropriate equine handling skills, yet within the literature it is commonly reported that time allocated for practical classes often suffers due to time constraint pressure from other elements of the curriculum. The effect of this pressure on animal handling teaching time is reflected in the self-reported low level of animal handling competency, particularly equine, in students with limited prior experience with horses. This is a concern as a naive student is potentially at higher risk of injury to themselves when interacting with horses. Additionally, a naive student with limited understanding of equine behaviour may, through inconsistent or improper handling, increase the anxiety and compromise the welfare of these horses. There is a lack of literature investigating the welfare of horses in university teaching facilities, appropriate handling procedures, and student safety. This article focuses on the importance for students to be able to interpret equine behaviour and the potential consequences of poor handling skills to equine and student welfare. Lastly, the authors suggest a conceptual model to optimise equine welfare, and subsequently student safety, during practical equine handling classes.

## 1. Introduction

Since the Classical Greek period, equitation has often been considered an art [1]. This romantic cultural perception of horse riding can, however, conflict with the practical considerations of risk [2]. Horseback riding is reported to be more dangerous than rugby, football, skiing and motorcycle riding [3,4,5] and the risks associated with equine-related activities are commonly accepted and well documented [2,6,7]. Many of the accidents and injuries to riders or handlers are often due to breakdowns in human-equine communication [6].

Horses are generally considered to be unpredictable, fearful and flight-wired and these are the traits linked to accidents involving people [8]. It has been proposed that the risk of injuries may be higher amongst people with low levels of knowledge of horse behaviour, as they may not be able to anticipate unwanted, but natural, equine behaviours [2]. As up to half of all equine related injuries take place during activities other than riding [9], it is likely that veterinary and animal science students may be a high risk group. Often these students handle horses with little previous experience and understanding of horse behaviour. This paper will focus on the use of horses for practical veterinary and animal science teaching purposes. The paper will also discuss the importance of students to be able to interpret equine behaviour and arousal levels and the potential consequences to student and equine welfare when this ability is lacking.

## 2. Equine Handling Classes

The majority of veterinary students come from an urban background with little, or no, previous animal handling experience [10,11,12]. In one survey, 60% of veterinary science and veterinary nursing students were from cities, 12% from large towns 10% from towns or villages and only 18% were from rural areas [11]. Due to a lack of previous exposure to animals, particularly large farm animals, veterinary science degrees have to focus on teaching basic animal behaviour and handling in combination with clinical examinations and procedures.

There is consensus across veterinary schools that the teaching of animal handling skills is a vital part of a veterinary degree [13,14,15,16,17,18,19]. Horses have been considered to be the most dangerous of all the animals that students have to learn how to handle appropriately [20], possibly because of their size and innate flight response. Across veterinary schools in Australasia, it appears that basic equine and other animal handling skills are mostly taught within the first year of the degree programme, with some universities reinforcing these skills throughout the programme [15,19]. The objectives of practical equine handling classes varies between universities, but many aim to teach students basic handling skills such as how to approach and restrain a horse with a halter and/or anti-rearing bit, lift fore and hind legs, groom the horse, apply and remove rugs and apply physical restraints, such as a nose twitch [13,14,15,16,17,18,19]. The practical equine handling classes are often delivered or taught within the first 2 years of a veterinary degree but the total duration of time allocated varies widely between universities, with reports ranging from 2 h to month-long full-time placements [13,15,16,17,18,19,20]. Good animal handling skills have been defined as general knowledge of the animal’s needs, practical experience in the care of the animal and the ability to quickly identify changes in behaviour, health or performance and to address those appropriately [21]. Based on this definition, it can be assumed that good equine handling skills require a considerable amount of time to master and are not taught in merely a few hours.

Providing learning opportunities in animal handling can be difficult and the time allocated has been reported to suffer due to pressure from other elements of the curriculum and due to financial constraints [13,21]. To combat this, a number of universities provide the opportunity for students to spend a few weeks, in the first two years of their degree, gaining practical equine experience extramurally [13,15,16,17]. Nevertheless, it is questionable whether, in some cases, only a few hours of practical teaching before partaking in extramural work experience is sufficient to prepare students to handle horses in a safe and effective manner. Austin et al. [17] reported that even after two 1.5 h practical equine handling sessions 38% of students failed their first attempt at a (basic) practical equine handling exam. Furthermore, the supervision and experience gained extramurally is likely to be inconsistent between placements and whether the students learn adequate safe and ethical horse handling skills is unknown. It is hence possible that competency between students may vary significantly, and be lacking, even after extramural work experience has been completed. Students at one university questioned the efficiency of the basic animal-handling skills being taught in the veterinary science programme and suggested a more structured approach be implemented [12].

The learning objectives for many equine handling classes appear strongly focused on physical handling and restraint [17,18,20], with lesser focus on observing the arousal state of the horse. Yet, appropriate restraint of all animals during veterinary and husbandry procedures depend on the ability to accurately assess the animals’ affective state. Cawdell-Smith et al. [13] suggest that students need to obtain more basic instruction in animal behaviour and handling, rather than an emphasis on manipulation and restraint, which is most likely only suitable for experienced students. Students need to develop their skills of observation and, as stated by McGreevy [21], “they need to develop a better understanding of animal behaviour and handling before they can become safe”. The welfare of all animals used in animal handling classes is also of critical importance and students need the skills to assess both the physical wellbeing and the arousal state of the animals they work with.

In many universities students are assessed on their animal-handling skills at various time points. The assessments often occur during the practical handling classes [13,20] and for many students this coincides with the first time they handle a particular species or perform a procedure. It would perhaps be useful to re-assess students throughout the veterinary program to ensure that their handling skills are improving. Some veterinary schools have already taken this approach, with students having to pass a barrier exam in animal handling at the end of their third year of study, prior to the start of the clinical teaching phase of the degree [13]. This not only achieves a threshold of minimum competency but provides an opportunity to reinforce the skills learnt earlier in the programme. Data on the efficacy of the different approaches, and level of repetition required to achieve desired minimum competencies are urgently required to help optimise equine handling experiences.

## 3. Is a Naive Handler at a Higher Risk of Injury?

A study investigating veterinary and equine students’ ability to assess the affective state and arousal of horses reported that less than half of the students correctly described the affective state of the horses [22]. Many students, especially those with less previous horse experience, failed to identify negative affective states and heightened arousal. This is perhaps not surprising as studies show that horse owners struggle to correctly identify certain behaviours and affective states [23]. An inability of veterinary students to accurately assess horse behaviour such as anxiety could create breakdowns in human-horse communication and subsequently pose a safety risk for the students [6]. Although protocols are in place in many universities with equine educational programs to ensure the safety of students and staff, accidents are still common. The most common injuries sustained by veterinary and animal science students during handling classes were inflicted by hind limb kicks, bites or by horses stepping on feet or ankles [22]. Many injuries occurred while standing near a horse (26.1%), performing a handling or husbandry procedure (21.7%) or performing a non-invasive physical examination procedure (17.4%). These data indicate a lack of awareness of the horse’s state of arousal and safe positioning around the horse by students. Injuries caused by the horse kicking or biting suggest an inability of the students to assess the affective state of the horse and subsequently respond appropriately when the horse is displaying anxious and reactive behaviours. Riley et al. [24] reports that 52% of students believed their injury was caused as a result of resistance by the horse to handling. However, inexperience and inattention was cited by students as being responsible for 39% and 30% of accidents, respectively [24]. These results further suggest a lack of experience or ability to observe and rapidly respond to behavioural cues displayed by the horse. Of injured students, 30% believed that the incident occurred because the horse was fearful or distressed. Perhaps some of these injuries could have been prevented if the students were better able to identify subtle cues from the horse, including changes in its arousal levels, and consequently moderate their behaviour and handling to attenuate this increased arousal level. The subtle, complex and rapidly changing nature of many equine behavioural cues may be difficult for a naive handler to process and correctly act upon. Gronqvist et al. [22] reports an association between previous horse experience and ability to assess the affective state of horses. These results imply that the ability to identify the subtle cues of equine behaviour improve over time and can hence be learnt.

## 4. The Welfare of Animals Used in Teaching

Live animals are a key aspect of animal handling. The sole use of virtual reality, simulators or models is not practical for teaching good animal handling skills [25,26]. With the use of live animals, however, comes the responsibility of treating them ethically and in accordance to the law. The Cruelty to Animals Act passed in Britain in 1876 was the first national law to regulate animals in research, testing and teaching. This bill was the first of its kind passed by any legislature in the world and provided a central governing body to review and approve the use of animals in research. Similar bills were quickly adopted in other countries and numerous enhancements have taken place since. Perhaps the most significant enhancement has been the principles universally acknowledged as the “3Rs”. The 3Rs stand for replacement, reduction and refinement [27] and form the foundation of modern research and teaching practices involving animals. Many veterinary schools strive to promote the 3R’s principle [28], however the complexity of equine behavior often restricts the efficacy of replacement of horses with simulations and veterinary schools thus reduce welfare cost by attempting to optimize the teaching experience and therefore focus on the reduction and refinement aspect of the 3R’s.

Today, most countries have laws governing the use of animals in teaching. Although there is some variation in the statutory requirements, the laws largely focus on the physical health and behavioural needs of the animals and to alleviate any pain and distress. Furthermore, in many countries, organisations using animals must follow an approved code of ethical conduct, which sets out the guidelines and procedures to be followed by the organisation and its animal ethics committee. Good animal welfare is the core of veterinary medicine, yet no universal definition or measure of welfare currently exists. Broom (1986) described animal welfare as the state of an animal in regards to its attempts to cope with its environment [29]. The health of the animal is the foundation of good welfare [30], although animal welfare does not just refer to the animals’ physical state [31]. The animals’ psychological state and ability to express natural behaviours are also vital to good welfare [32]. Animal welfare has been described as complex and multifactorial, therefore proving challenging to define and assess [33]. Animal welfare assessments thus need to be holistic, rather than focus solely on one aspect, such as the animal’s physical state alone [31]. The welfare of an animal is a reflection of its affective state, according to The Five Domains [34]. The Five Domains model was initially developed to evaluate welfare compromise in animals used in research, teaching and testing [35]. This model is used to recognise compromise in the four physical domains (nutrition, environment, health and behaviour) and in one mental domain, which is the animal’s affective experiences and reflects the animal’s overall welfare state [34]. In order to maximise welfare, it is thus important for veterinary students to learn how to assess the horse’s affective state, in addition to the physical state [34,36]. It is therefore proposed that the framework of such a model should be more methodically incorporated in the theoretical and practical veterinary teaching curriculum.

## 5. Does an Inability of a Naive Handler to Read Behaviour Have Implications for Equine Welfare?

It has been strongly emphasised that daily human-equine interaction greatly influences the way horses perceive humans and the consequential relationship [37,38]. Many common husbandry procedures and circumstances in which veterinary students handle horses may be perceived as an aversive stimuli by the horse, e.g., restraint, diagnostic examinations and practising clinical procedures. Many aversive stimuli paired with little positive reinforcement increase the risk of horses entering negative affective states, which could have an impact on the horses’ overall welfare [39,40]. There is significant opportunity for the amplification of the aversive stimuli in the hands of many students that may be naive, or have limited ability to interpret horse behaviour and competently handle horses in any but the most favourable of situations. Overmier and Wielkiewicz [41] reported that if human signals become inconsistent, animals may exhibit behaviours indicative of extreme arousal and reactivity. Incorrectly applying aversive stimuli in horse training has been proposed to compromise horse welfare [42]. Horses used for veterinary teaching are frequently handled by a large number of students with varying skill levels. It is thus presumable that there are inconsistencies in the way horses are handled by veterinary students, which could lead to increased arousal levels and anxiety and compromised welfare in these horses. It is, therefore, likely that the unavoidable use of aversive stimuli, coupled with a lack of consistent human signals during human-equine interactions in teaching, induces equine anxiety. In addition to reduced welfare for the horse, repetitive exposure to negative human-equine interactions puts the handlers at risk of serious accidents [43]. To minimise the potential negative effects that handling classes may have on horse welfare, it is suggested that horses are frequently rotated between classes to minimise their exposure to aversive stimuli. Teaching learning theory to students could be combined with horse handling classes in an attempt to introduce the use of positive reinforcement and minimise the use of aversive stimuli.

## 6. The Balance between Workload and Experience

The psychology and business management literature describe the concepts of flow and optimal stress, when the appropriate balance between workload and experience is achieved [44]. Using this concept individuals achieve optimal flow when the frequency and complexity of the tasks are appropriate for the level of skill and competency of the individual. Excessive stress occurs when, the frequency, the complexity, or the interaction of the two, exceed the individuals capability. Optimal or acceptable stress is when the individual is operating within the zone of flow; sufficient workload to maintain interest, without providing excessive stress. This concept could be applied to the use of horses in practical teaching of veterinary and animal science students.

**Figure 1:**
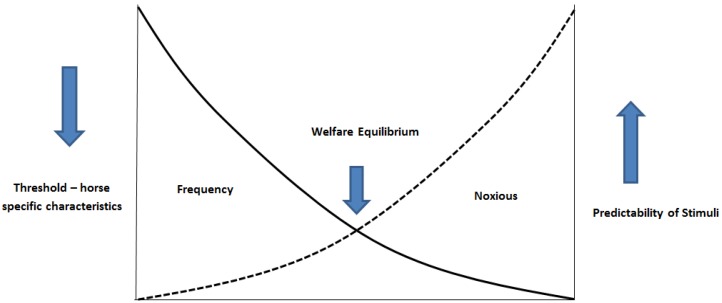
Schematic representation of the interaction of the variables frequency of use, noxiousness of the interaction and horse threshold traits on the welfare state of the horse during veterinary practical classes. The initial set point (the respective intercepts of frequency and noxiousness on their respective *Y* axis) is horse specific or situation (handler and/or environment) specific, and thus moderates the slope and respective intersection point for welfare equilibrium for a specific horse. The slopes of the respective lines are also moderated by the predictability of the stimuli.

To reduce the welfare cost to the horse, horse anxiety and the associated risk of handler injury, the optimal balance between the averseness of the stimuli, frequency of stimuli and predictability of the stimuli needs to be achieved for each horse (Figure 1). The welfare equilibrium is achieved at the intersection of the frequency of use and noxiousness of the stimuli. Within the model we have portrayed frequency and noxiousness as curvilinear relationships, rather than a simple linear slope, to reflect the biology of the system. For each horse and situation there will be a threshold in which the adversity of the frequency and noxiousness of stimuli no longer has a simple additive effect. The initial set point (the respective intercepts of frequency and noxiousness on their respective *Y* axis) will be horse specific or even situation (handler and/or environment) specific, and thus also moderates the slope and respective intersection point for welfare equilibrium for a specific horse. The slopes of the respective lines are also in turn moderated by the predictability of the stimuli.

For horses used in teaching facilities, student interaction would be considered the stimuli. The veterinary procedure (e.g., venipunctures or rectal palpations) the students practice would determine the averseness of the stimuli. Within veterinary teaching programmes there are limited opportunities to modify the procedures horses are used for, and class size restrictions limit our ability to modify the severity and frequency of horse and student interaction. The greatest ability to reduce welfare cost, anxiety and exposure (risk) to injury is to moderate the slope of the lines through greater consistency and decreased averseness of the human-horse interaction.

## 7. Optimising Welfare in a Teaching Environment

The model proposed in Figure 1 could be further refined with the concept of exposure to teaching and class specific moderators that either dampen or accentuate the averseness of the stimuli (in essence modifiers of the slope). One such moderator is the previous learning experience of individual horses. The previous history of punishment or reinforcement associated with a specific stimulus will affect operant and emotional responses to similar cues in the future and the horse will form a negative association or fear response [44,45]. Because of the mechanisms of learning, once a fear response has been formed it is difficult to erase [46]. The fear response can be modified but spontaneous reoccurrence is likely [47,48,49]. Steps should thus be taken to reduce exposure to negative environments and effective states to reduce the risk of formation of negative associations. Particular care should be focused on optimising student-horse experience through providing positive experiences which will provide longevity and a reduction in welfare cost. Moderators, such as previous learning history and predictability of the stimulus, will act directly on the point of intersection and on the level of arousal (the perceived noxiousness of the stimuli). Anecdotal observations by the authors have indicated that, even with large class sizes, mild changes in the predictability of the student-horse interaction (i.e., slower and more obvious interactions) have reduced overt signs of anxiety in teaching horses, even though the averseness of the stimuli (rectal palpation) remained consistent.

### 7.1. Preventing Horse Isolation

Horses are social creatures and social isolation can therefore generate an anxiety state [50,51]. As such, social isolation may contribute to the noxiousness of the interaction. It is hence plausible to suggest that modification of the environment to meet the social needs of the horse would reduce the threshold for anxiety. Preliminary data by our group indicate that social isolation may over-ride perception of even moderate (noxious stimuli) pain (Reid et al in prep). Thus given the horse’s strong drive for social interaction the role of social isolation on moderating the horses experience during practical teaching classes should not be underestimated as a source of anxiety and potential risk. Establishing a teaching environment that meets horses’ social needs (e.g., the sight of other horses) could potentially reduce the risk of student injury and should be an integral part of any teaching environment.

### 7.2. Inherent Characteristics of Horses and Humans

Another moderating factor on the slope and the intersection of the lines for frequency and averseness of the stimuli are the inherent characteristics of teaching horses. Horses used for teaching purposes are often chosen based on their sedate characteristics and ease of handling [17,19,20]. While individual differences exist [52,53,54], it has been shown that horses’ behavioural responses to challenges can be modified by both external (e.g., type of work) and internal (e.g., breed) factors [38,55]. Older horses are often selected for teaching purposes [12], but research suggests that more attention may need to be placed on breed and management strategies of these horses. When exposed to a number of temperament testing procedures, breed and housing conditions of horses have been shown to have a greater influence on behaviour, while sex and age of the horse have lesser influence [56]. Riding school horses managed in individual boxes reacted more strongly when released in an arena, especially if a novel object was present, than horses managed as a herd on pasture [53]. These riding horses were more prone to express “high locomotory components”. While release into an arena is a different model to the common use of horses in veterinary practical teaching, the greater predisposition to high locomotor components does suggests a reduction in the individual horse’s threshold for the flight response. The hardwired flight behaviour in the horse is a consistent theme in the risk associated with horse handling and thus reduction in the flight response threshold will increase risk. These horses may then require physical or chemical restraint to attenuate this risk, which may not have been necessary with modification of the management system. For safety reasons, naive students may benefit from initially only interacting with horses of a more sedate character. Graded exposure techniques would allow nervous students to gradually habituate to handling horses with different levels of reactivity and previous handling experiences [57,58]. As the students progress with their handling skills they may benefit from experience with horses of a more reactive nature to prevent complacency and prepare them for the wide range of horses they are likely to encounter in their career. The ability of students to interpret the behavioural and arousal state of horses increases with experience [22], however, increased exposure may lead to less vigilance as inattention was cited by veterinary students as being responsible for 30% of horse-handling accidents [24].

The inherent characteristics of the student handler are another moderating factor on the slope and the intersection of the lines for frequency and averseness of the stimuli. Human emotional states and attitudes towards animals influence human-animal communication [59,60]. Within the context of commercial livestock industries there is a significant body of evidence demonstrating the advantage of training programmes improving stock person handling skills and the subsequent reduction in fear and stress responses in livestock [21]. Thus an active training programme for students based around ethology and learning theory should significantly reduce the welfare cost to the teaching horses. Within the literature a number of studies provide support for such an approach that not only provides a greater opportunity for positive human-horse interactions, but has an indirect effect on increasing handler confidence and a positive attitude towards the horse. Confidence, irrespective of handling experience, has been reported to alter the human-horse interaction [61,62]. Horses were reported to exhibit less ear movement, and more frequently had a forward ear position (suggesting confidence, relaxation, and interested in surroundings [45]), when led by veterinary students with a positive attitude towards horses [62].

A nervous handler may also provide an environment with a heightened state of arousal, which primes the horse to respond to potential danger as the startle response threshold is reduced [61,63]. Students who are both inexperienced and nervous around horses may thus have the greatest negative effect on horse welfare as, in addition to an increased heart rate [61], the students are also more likely to send inconsistent signals. These individual human characteristics should be considered in the model, and should be a focus of training during animal handling practicals, as they act directly on the point of intersection and the welfare equilibrium.

### 7.3. Actions Points and Empirical Validation of the Model

At present the model proposed is purely a conceptual model. Further refinement of this model requires the application of metrics to transform this from a conceptual framework to an empirical model. However, despite the current lack of metrics there are a number of practical steps that can be implemented within the practical teaching environment, until such data can be collected and the model has been verified and tested. These practical steps include

1. Predictability: Ensuring student awareness of equine ethology and the horse’s ecological niche prior to the start of practical session, as this information should directly translate to a greater predictability of human-horse interactions. A basic ethogram of equine arousal levels is needed to ensure a simple, practical and easy to use method to teach students predictable, responsive interactions with horses and subsequently reduce the risk of injury to themselves while improving horse welfare.
2. Horse specific characteristics and adverseness of stimuli: Exposure of students to learning theory early in the curriculum prior to horse handling. This should provide awareness that each human-horse interaction has a modifying effect (good or bad). Punishment and unclear or inconsistent signals may heighten the horse’s arousal and negative affective state and lead to confusion, conflict or fear behaviors which may become a safety issue for the handlers [49]. Teaching would instead emphasize the ability to use positive reinforcement techniques (addition of an attractive stimulus to increase the likelihood of a desired response) as a means to shape and increase desired behaviours and reduce the chance of negative (fear related) associations forming.
3. Consider the inherent characteristics of individual horses, such as age, temperament and previous learning history.
4. Prevent additional anxiety caused by social isolation by allowing conspecifics within view of the subject horse.

To provide empirical validation of the model data need to be collected in the teaching classes across different teaching environments. For a number of criteria such as noxiousness and predictability there is a need for the development of a simple and practical system to quantify these with sufficient precision.

## 8. Conclusions

The challenge for veterinary programmes is to help students develop appropriate handling skills by identifying the optimal model to teach students to assess horse behaviour and its application to animal handling skills. Teaching stockmanship skills has already been shown to improve attitude of cattle handlers and enhance the welfare and productivity of cattle [48]. It is thus probable that similar benefits could be gained from improved equine handling skills. An enhanced quantification of “skill” is needed, as currently there does not appear to be a consensus in the literature on what are good, acceptable or poor equine handling skills. In most veterinary schools teaching horses provide the ideal mechanism to develop horse handling skills. However, consistent exposure to naive or poor handling provides an opportunity for compromised welfare of the teaching horses.

The conceptual model proposed provides the first step towards identifying the variables that contribute to compromised welfare and mechanisms to mitigate these. Collection of empirical data during teaching practicals will allow validation of the model and quantification of the benefit of changes in management.
